# Formation mechanism of glandular trichomes involved in the synthesis and storage of terpenoids in lavender

**DOI:** 10.1186/s12870-023-04275-y

**Published:** 2023-06-08

**Authors:** Yanan Zhang, Di Wang, Hui Li, Hongtong Bai, Meiyu Sun, Lei Shi

**Affiliations:** 1 Key Laboratory of Plant Resources, Institute of Botany, Chinese Academy of Sciences, Beijing, 100093 China; 2 China National Botanical Garden, Beijing, 100093 China; 3 University of Chinese Academy of Sciences, Beijing, 100049 China

**Keywords:** Lavender, Volatile organic compounds (VOCs), Glandular trichomes (GTs), Formation mechanism

## Abstract

**Background:**

Lavender (genus *Lavandula*, family Lamiaceae) is an aromatic plant widely grown as an ornamental plant. The chemical composition of lavender is characterized by monoterpenoids, sesquiterpenoids, and other compounds, which are primarily synthesized and stored in epidermal secretory structures called glandular trichomes (GTs). Volatile organic compounds (VOCs) are responsible for the aroma characteristics of plant oil that drive consumer preference. Aroma is usually regarded as a characteristic trait for the classification of aromatic plants. Interestingly, VOCs are synthesized and stored in GTs. Lamiaceae species such as purple perilla, peppermint, basil, thyme, and oregano usually possess two types of GTs: peltate glandular trichomes (PGTs) and capitate glandular trichomes (CGTs). But the development process of PGTs in lavender has been reported in only a few studies to date.

**Results:**

In this study, we identified and quantified the VOCs in four lavender cultivars by headspace-solid phase micro extraction-gas chromatography mass spectrometry (HS–SPME–GC–MS). A total of 66 VOCs were identified in these four cultivars, the most prominent of which were linalyl acetate and linalool, and flowers were the main site of accumulation of these VOCs. Here, we examined the developmental process of PGTs, including the formation of their base, body, and apex. The apex cells contained secretory cavities, which produced VOCs. Based on the reference genome sequence of the lavender cultivar ‘Jingxun 2’, several R2R3-MYB subfamily genes related to GT formation were identified. These results will guide the engineering of GTs and molecular breeding of lavender for improving the VOC content.

**Conclusions:**

In this study, we identified the VOCs in four lavender cultivars. We analyzed the formation of GTs, and compared the number and diameter size of PGTs among four lavender cultivars. Additionally, we identified four candidate genes belonging to the R2R3-MYB family.

**Supplementary Information:**

The online version contains supplementary material available at 10.1186/s12870-023-04275-y.

## Background

**Fig. 1:**
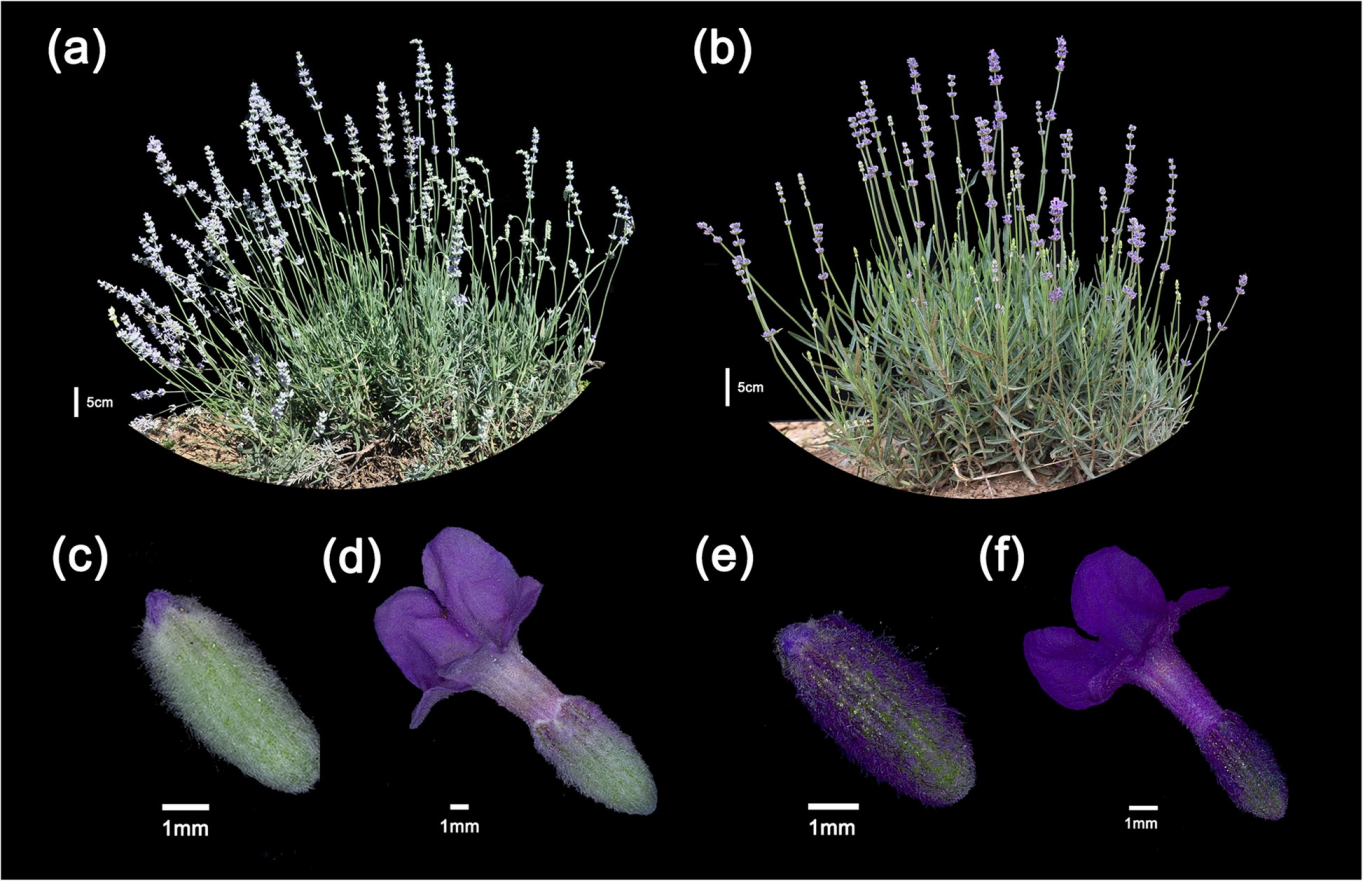
Photographs of ‘Jingxun 1’ and ‘Jingxun 2’ plants and theirs flowers at different stages. **a** ‘Jingxun 1’. **b** ‘Jingxun 2’. **c**, **d** Flower bud (**c**) and flower (**d**) of ‘Jingxun 1’. **e**, **f** Flower bud (**e**) and flower (**f**) of ‘Jingxun 2’

The genus *Lavandula* of the Lamiaceae family comprises approximately 39 species. Among the *Lavandula* species, *L. angustifolia*, *L. latifolia*, and their natural sterile hybrid *L.* × *intermedia* are cultivated worldwide and used to manufacture perfumes, cosmetics, pharmaceuticals, and more recently, aroma therapy products, among which lavender is also as an aromatic plant widely grown as an ornamental plant [1–4]. ‘Jingxun 1’ and ‘Jingxun 2’ are improved lavender varieties selectively bred by the Institute of Botany, Chinese Academy of Sciences (CAS) using flower yield, essential oil yield, and other factors as evaluation indexes (Fig. 1a and b). The sepal color of ‘Jingxun1’ is lighter than that of ‘Jingxun 2’ (Fig. 1c–f). Additionally, the essential oil of ‘Jingxun 2’ is characterized by high levels of linalyl acetate and linalool and low amounts of camphor, and is the most common additive in many over-the-counter medicines and cosmetic products in China [5].

Lavender is also an important spice appreciated for its aroma. Volatile compounds of lavender contribute significantly to its aroma [6]. Volatile organic compounds (VOCs) are responsible for the aroma characteristics of plant oil that drive consumer preference. VOCs play several biologically important roles, such as acting as cues between plants and other organisms (herbivores, pathogens, pollinators, parasitoids, and plants) [7]. Given their roles in plant survival and environmental adaptation, VOCs are of major interest in chemical ecology. The main groups of volatiles present in aromatic plants are terpenes (such as monoterpenes and sesquiterpenes), aldehydes, alcohols, ketones, esters, and hydrocarbons. Therefore, there is much interest in the VOCs of lavender.

Trichomes are specialized outgrowths of epidermal cells with diverse structures and functions, and could be categorized based on their cell number (unicellular and multicellular) and secretory ability (glandular and non-glandular) [8, 9]. Although unicellular trichomes have no secretory function, they protect plants against natural hazards, such as herbivores, ultraviolet (UV) irradiation, pathogen attack, and excessive transpiration, and facilitate seed dispersal [10–14]. The model plant *Arabidopsis thaliana* possesses typical unicellular non-glandular trichomes (NGTs). Glandular trichomes (GTs) can synthesize, store, and secrete large amounts of bioactive metabolites, such as terpenoids, phenylpropanoids, flavonoids, alkaloids, and acyl sugars [15]. Five plant species including tomato (*Solanum lycopersicum*), cucumber (*Cucumis sativus*), sweet wormwood (*Artemisia annua*), tobacco (*Nicotiana tabacum*), and cotton (*Gossypium hirsutum*) have been the key plant materials for GT research in recent years [16]. Members of the Lamiaceae family, such as purple perilla (*Perilla frutescens*) [17], peppermint (*Mentha* x *piperita*) [18], basil (*Ocimum basilicum*) [19], thyme (*Thymus quinquecostatus*) [20], and oregano (*Origanum vulgare*) [21], usually possess two kinds of trichomes, namely, peltate glandular trichomes (PGTs) and capitate glandular trichomes (CGTs).

In this study, we determined the contents of VOCs in the leaves and flowers of four lavender varieties by HS–SPME–GC–MS. Additionally, we examined the fresh leaves and flowers of all four varieties by fluorescence microscopy and scanning electron microscopy, and counted the number of GTs. Moreover, we elucidated the ontogeny of PGTs in this study. Based on the reference genome sequence of ‘Jingxun 2’ [5], several R2R3-MYB subfamily genes related to GT formation were identified in lavender. These results will guide the engineering of GTs and the molecular breeding of lavender.

## Results

### Identification of VOCs in lavender varieties

**Fig. 2:**
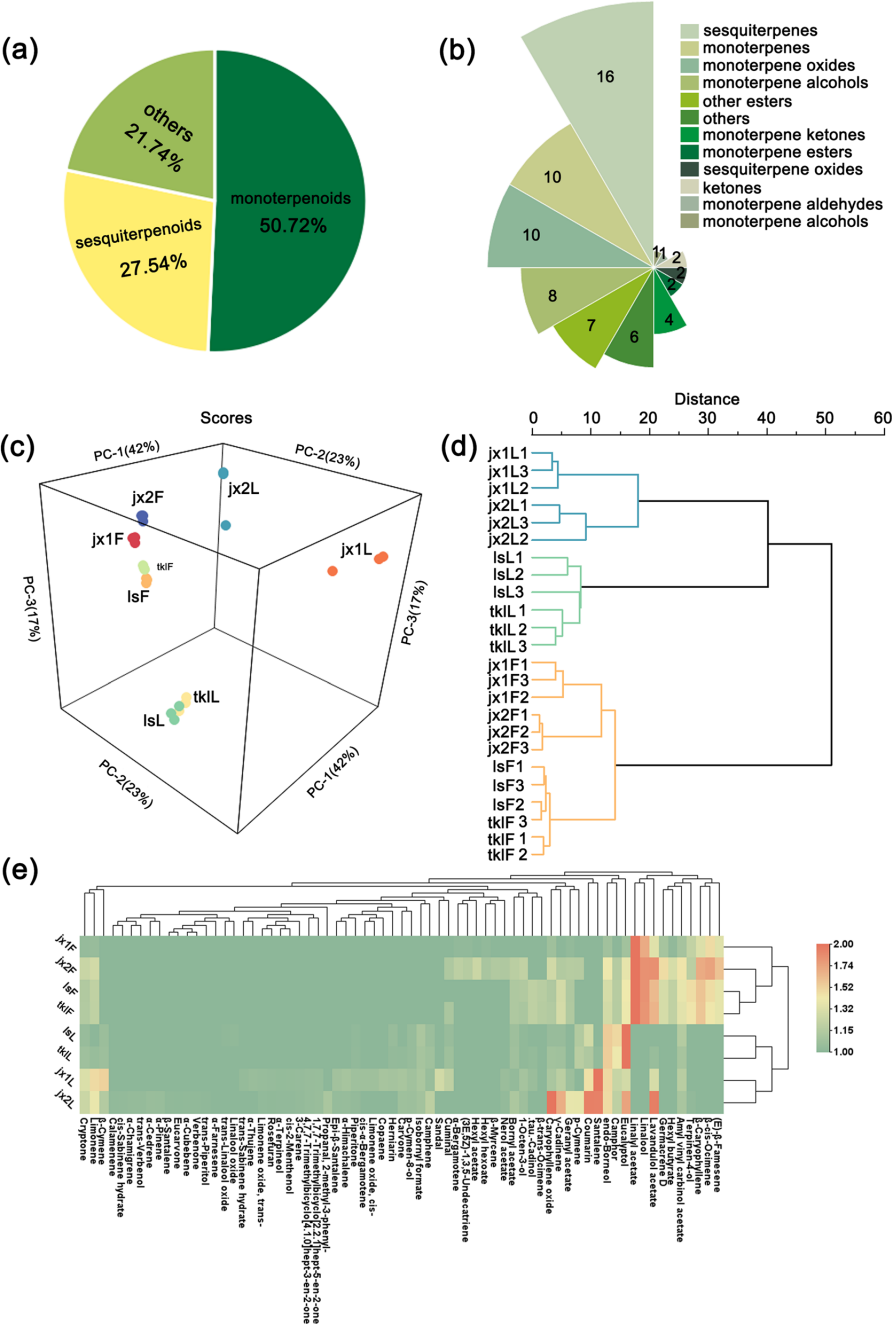
Volatile organic compounds (VOCs) identified in four lavender varieties by HS–SPME–GC–MS. **a** Classification of VOCs. **b** Classification of 66 VOCs into 12 classes. **c–e** PCA score map (**c**), dendrogram (**d**), and heatmap (**e**) of VOCs

A total of 66 VOCs were identified in four lavender varieties by HS–SPME–GC–MS. The VOCs were mainly divided into two categories: monoterpenoids and sesquiterpenoids (Fig. 2a). Monoterpenes (10) and monoterpene oxides (10) were present in equal number, and together accounted for 14.49% of all monoterpenoids. In addition, monoterpene alcohols (8) accounted for 11.59% of all monoterpenoids, while the other monoterpenoids included monoterpene esters (2), aldehydes (1), and ketones (4). Sesquiterpenes (16) accounted for the vast majority of sesquiterpenoids, and no esters, aldehydes, and ketones were identified in this group (Fig. 2b). Principal component analysis (PCA) is a technique used to determine the general metabolic differences between groups and within a group. Therefore, we performed PCA to further classify these samples (leaves and flowers of ‘Jingxun 1’, ‘Jingxun 2’, ‘Luoshen’, and ‘Taikonglan’) (Fig. 2c). The results showed that the first principal component (PC1) explained 42.00% of the total variance and separated samples (a sample represented a tissue type). The second principal component (PC2) explained 23.00% of the total variance. All three replicates of a sample grouped together, and significant differences were detected. The VOCs extracted from the flowers of the four lavender varieties were closely related, while those extracted from leaves showed large variation. Overall, these results revealed that the type of VOCs differed among the four lavender cultivars as well as among the different tissues of a given cultivar.

Hierarchical clustering analysis of the VOCs revealed three main clusters (Fig. 2d). The leaf-derived VOCs of ‘Jingxun 1’ and ‘Jingxun 2’ clustered together into one group, while those of ‘Luoshen’ and ‘Taikonglan’ clustered into another group. By contrast, VOCs identified in the flowers of all four lavender cultivars formed a separate group. These results were in accord with the results of PCA, indicating that the VOCs found in the flowers and leaves of lavender cultivars are critical for elucidating the basis of aroma variation among lavender cultivars. Accordingly, the details of differentially expressed VOCs were analyzed in this study.

The chemical composition of each of the four lavender cultivars is summarized in Table 1. Linalyl acetate and linalool were the most prominent and characteristic VOCs found in flowers. Linalyl acetate was present in high amounts in ‘Jingxun 2’, ‘Luoshen’ and ‘Taikonglan’ flowers (85.16, 93.19, and 88.28 mg/g, respectively) but in a low amount (4.01 mg/g) in ‘Jingxun 1’ flowers. Interestingly, in ‘Jingxun 2’ and ‘Luoshen’ leaves, the content of each VOC, regardless of its type, was below 1.00 mg/g. Additionally, the contents of lavandulol acetate and β-caryophyllene were also relatively high. These results indicated that flowers are the main site of accumulation of VOCs (Fig. 2e).

**Table 1:** Details of terpenoids identified in the leaves and flowers of four lavender varieties

No	RT	RI	Terpenoid	‘Jingxun 1’ (mg/g)		‘Jingxun 2’ (mg/g)		‘Luoshen’ (mg/g)		‘Taikonglan’ (mg/g)	
				**Flower**	**Leaf**	**Flower**	**Leaf**	**Flower**	**Leaf**	**Flower**	**Leaf**
**1**	5.176	929	α-Thujene	-	0.12 ± 0.02	-	-	-	-	-	-
**2**	5.234	937	α-Pinene	-	-	-	0.02 ± 0.01	-	-	-	-
**3**	5.515	952	Camphene	-	0.12 ± 0.02	-	0.08 ± 0.04	-	0.08 ± 0.02	-	0.12 ± 0.02
**4**	6.111	980	1-Octen-3-ol	-	0.25 ± 0.04	2.25 ± 0.17	0.03 ± 0.01	1.31 ± 0.03	0.02 ± 0.00	0.96 ± 0.06	0.02 ± 0.00
**5**	6.465	991	β-Myrcene	-	-	1.25 ± 0.26	-	-	-	-	-
**6**	6.936	1010	3-Carene	-	0.09 ± 0.00	-	0.01 ± 0.01	-	-	-	-
**7**	6.975	1011	Hexyl acetate	-	-	2.95 ± 0.46	-	-	-	-	-
**8**	7.286	1023	β-Cymene	-	1.92 ± 0.93	-	0.06 ± 0.03	-	0.08 ± 0.00	-	0.10 ± 0.00
**9**	7.324	1025	p-Cymene	-	-	1.50 ± 0.00	0.16 ± 0.08	-	0.21 ± 0.01	-	0.29 ± 0.01
**10**	7.423	1030	Limonene	0.10 ± 0.04	1.75 ± 0.80	3.68 ± 0.19	0.16 ± 0.00	2.23 ± 0.54	-	2.16 ± 0.16	0.07 ± 0.03
**11**	7.503	1032	Eucalyptol	0.19 ± 0.07	0.87 ± 0.13	6.45 ± 0.27	0.30 ± 0.06	8.89 ± 0.69	1.77 ± 0.31	7.78 ± 0.52	2.12 ± 0.19
**12**	7.632	1037	β-cis-Ocimene	1.47 ± 0.22	-	34.56 ± 4.50	-	11.66 ± 0.52	-	9.15 ± 1.06	-
**13**	7.875	1049	β-trans-Ocimene	-	-	-	-	1.70 ± 0.03	-	1.57 ± 0.06	-
**14**	8.388	1066	trans-Sabinene hydrate	-	0.08 ± 0.04	-	-	-	-	-	-
**15**	8.419	1067	cis-Sabinene hydrate	-	-	-	0.01 ± 0.01	-	-	-	-
**16**	8.559	1074	Linalool oxide	-	-	-	-	-	0.02 ± 0.00	-	-
**17**	8.856	1084	Rosefuran	-	0.09 ± 0.00	-	0.01 ± 0.00	-	-	-	-
**18**	8.977	1086	trans-Linalool oxide	-	-	-	-	-	0.02 ± 0.00	-	-
**19**	9.305	1099	Linalool	2.43 ± 0.21	-	49.03 ± 6.03	-	51.97 ± 1.94	-	47.11 ± 2.57	-
**20**	9.571	1111	Amyl vinyl carbinol acetate	0.23 ± 0.03	0.48 ± 0.05	9.29 ± 1.87	0.05 ± 0.02	7.02 ± 0.58	0.23 ± 0.06	5.92 ± 0.06	0.16 ± 0.01
**21**	9.875	1122	cis-2-Menthenol	-	0.08 ± 0.07	-	0.01 ± 0.00	-	-	-	-
**22**	10.231	1134	Limonene oxide, cis-	-	0.18 ± 0.09	-	-	-	-	-	-
**23**	10.251	1136	trans-Verbenol	-	-	-	0.01 ± 0.01	-	-	-	-
**24**	10.303	1139	Limonene oxide, trans-	-	0.10 ± 0.01	-	0.01 ± 0.01	-	-	-	-
**25**	10.509	1145	Camphor	-	0.69 ± 0.02	1.49 ± 0.51	0.08 ± 0.04	1.62 ± 0.24	0.80 ± 0.10	1.55 ± 0.09	0.74 ± 0.06
**26**	11.075	1167	endo-Borneol	0.18 ± 0.06	1.78 ± 0.30	7.84 ± 0.89	0.20 ± 0.09	4.30 ± 0.40	0.90 ± 0.10	3.78 ± 0.28	1.03 ± 0.05
**27**	11.208	1174	(3E,5Z)-1,3,5-Undecatriene	0.04 ± 0.00	-	1.54 ± 0.10	-	-	-	-	-
**28**	11.398	1177	Terpinen-4-ol	0.54 ± 0.06	-	1.85 ± 0.36	0.01 ± 0.00	11.29 ± 0.63	-	9.52 ± 0.84	-
**29**	11.485	1183	p-Cymen-8-ol	-	0.21 ± 0.00	-	0.03 ± 0.01	-	0.05 ± 0.00	-	0.06 ± 0.01
**30**	11.581	1184	Cryptone	0.08 ± 0.03	1.02 ± 0.01	2.78 ± 0.20	0.11 ± 0.05	1.33 ± 0.10	0.06 ± 0.01	1.36 ± 0.14	0.11 ± 0.03
**31**	11.756	1191	Hexyl butyrate	0.04 ± 0.00	-	4.26 ± 0.46	-	2.25 ± 0.01	-	2.24 ± 0.11	-
**32**	11.76	1192	α-Terpineol	-	0.07 ± 0.00	-	0.01 ± 0.01	-	-	-	-
**33**	12.212	1209	trans-Piperitol	-	-	-	0.01 ± 0.00	-	-	-	-
**34**	12.269	1211	Verbenone	-	-	-	0.01 ± 0.00	-	-	-	-
**35**	12.634	1224	Eucarvone	-	-	-	0.01 ± 0.00	-	-	-	-
**36**	12.805	1232	Isobornyl formate	-	0.30 ± 0.01	-	0.04 ± 0.02	-	0.17 ± 0.03	-	0.16 ± 0.03
**37**	13.089	1239	Cuminal	-	0.57 ± 0.09	1.13 ± 0.10	-	-	0.06 ± 0.00	0.97 ± 0.12	0.11 ± 0.02
**38**	13.192	1242	Carvone	-	0.28 ± 0.02	-	0.03 ± 0.01	-	-	-	0.04 ± 0.01
**39**	13.481	1253	Piperitone	-	0.19 ± 0.00	-	0.01 ± 0.00	-	0.02 ± 0.00	-	0.03 ± 0.01
**40**	13.546	1257	Linalyl acetate	4.01 ± 0.55	-	85.16 ± 5.28	-	93.19 ± 5.16	-	88.28 ± 0.07	-
**41**	14.344	1285	Bornyl acetate	-	0.07 ± 0.00	1.81 ± 0.10	0.05 ± 0.03	1.14 ± 0.13	0.09 ± 0.01	1.12 ± 0.04	0.12 ± 0.02
**42**	14.42	-	Lavandulol acetate	0.95 ± 0.02	0.94 ± 0.05	49.81 ± 3.85	0.36 ± 0.28	23.28 ± 1.83	-	25.28 ± 2.65	0.07 ± 0.03
**43**	16.104	1351	α-Cubebene	-	-	-	0.01 ± 0.00	-	-	-	-
**44**	16.35	1364	Nerol acetate	-	0.13 ± 0.03	1.19 ± 0.11	0.01 ± 0.01	-	-	-	-
**45**	16.738	1376	Copaene	-	0.26 ± 0.07	-	-	-	-	-	-
**46**	16.841	1382	Geranyl acetate	0.06 ± 0.01	0.16 ± 0.01	1.57 ± 0.20	0.16 ± 0.03	1.20 ± 0.05	-	1.25 ± 0.15	-
**47**	16.894	1384	Hexyl hexoate	0.04 ± 0.00	-	0.90 ± 0.06	-	-	-	-	-
**48**	17.708	1411	α-Cedrene	-	-	-	0.02 ± 0.01	-	-	-	-
**49**	17.711	1415	cis-α-Bergamotene	-	0.18 ± 0.02	-	-	-	-	-	-
**50**	17.844	1420	Santalene	-	4.93 ± 0.11	-	0.36 ± 0.05	-	-	-	0.04 ± 0.01
**51**	17.871	1425	β-Caryophyllene	0.91 ± 0.05	-	29.44 ± 3.61	-	19.24 ± 0.60	-	17.56 ± 2.48	-
**52**	18.217	1435	α-Bergamotene	0.04 ± 0.00	-	1.93 ± 0.15	-	-	-	-	-
**53**	18.22	1441	Coumarin	-	1.61 ± 0.42	-	0.36 ± 0.07	-	0.43 ± 0.08	-	0.22 ± 0.10
**54**	18.471	1449	α-Himachalene	-	0.26 ± 0.07	-	0.02 ± 0.01	-	-	-	-
**55**	18.528	1448	Epi-β-Santalene	-	0.21 ± 0.01	-	0.02 ± 0.01	-	-	-	-
**56**	18.703	1457	(E)-β-Famesene	0.95 ± 0.06	-	18.60 ± 2.59	-	6.67 ± 0.66	-	6.49 ± 0.73	-
**57**	18.856	1462	β-Santalene	-	-	-	0.01 ± 0.00	-	-	-	-
**58**	19.395	1481	Germacrene D	0.23 ± 0.02	-	11.12 ± 1.70	-	4.22 ± 0.29	-	4.14 ± 0.60	-
**59**	19.961	1508	α-Farnesene	-	-	-	-	-	-	-	-
**60**	20.177	1513	γ-Cadinene	-	0.51 ± 0.02	1.33 ± 0.05	0.28 ± 0.07	3.94 ± 0.92	0.06 ± 0.01	4.02 ± 0.10	0.12 ± 0.05
**61**	20.269	1523	α-Chamigrene	-	-	-	0.01 ± 0.00	-	-	-	-
**62**	20.383	1528	Calamenene	-	-	-	0.01 ± 0.00	-	-	-	-
**63**	20.618	1538	Sandal	-	0.85 ± 0.04	-	-	-	-	-	-
**64**	21.835	1559	Caryophyllene oxide	0.16 ± 0.02	0.31 ± 0.09	3.60 ± 0.14	0.38 ± 0.01	1.20 ± 0.06	0.06 ± 0.02	1.37 ± 0.29	0.13 ± 0.03
**65**	23.115	1646	Cadinol	-	0.12 ± 0.01	-	-	2.13 ± 0.59	-	2.07 ± 0.28	0.05 ± 0.02
**66**	24.895	1732	Herniarin	-	0.25 ± 0.00	-	-	-	0.06 ± 0.02	-	0.04 ± 0.02

Values are reported as mean ± standard deviation of three parallel experiments; ‘-’ means not detected

*RT* Retention time, *RI* Retention indices

### Morphology and development of GTs in *L. angustifolia*

**Fig. 3:**
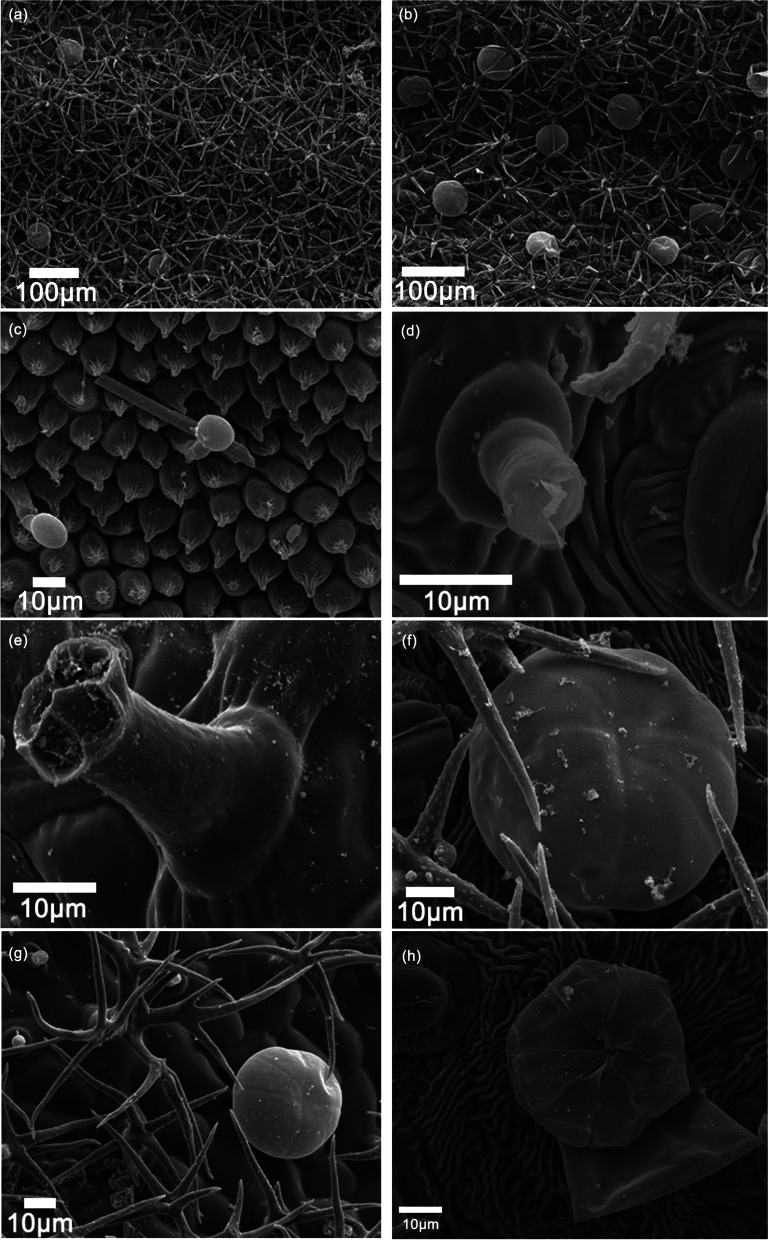
Scanning electron microscopy (SEM) analysis of the different types of trichomes found on different tissues. **a**, **b** Adaxial (**a**) and abaxial (**b**) surface of ‘Luoshen’ leaves. **c** Petal of ‘Luoshen’. **d, e** Abaxial surface of ‘Jingxun 2’ leaves. **f**, **g** Abaxial (**f**) and adaxial (**g**) surface of ‘Luoshen’. **h** Cuticle rupture of peltate glandular trichomes (PGTs)

The leaf surface was covered with three types of GTs: peltate, capitate, and NGTs (Fig. 3a–h). PGTs and NGTs were located on sepals (Supplementary Fig. S1) and on the adaxial and abaxial surfaces of leaves. However, only CGTs were found on the petals (Fig. 3c). Like the GTs in other Lamiaceae plants, PGTs in lavender cultivars were composed of three cell types which were base, stalk, and head [22, 23]. The head cell is responsible for the secretion of specialized metabolites; the stalk is the structure bearing the gland; and the base connects the stalk to the surrounding epidermal cells.

Stereomicroscopy analysis showed that the three types of GTs were not only found on leaves and flowers but were also distributed on stems (Supplementary Fig. S2). PGTs consisted of one stalk cell, one basal cell, and several multicellular heads, each one of which consisted of eight cell disk-shaped head and assumed a globular dome shape at maturity (Supplementary Fig. S3). In other words, they were constituted by a layer of basal cells, which gave continuity to the epidermis. CGTs were smaller than PGTs, and were composed of a basal cell, a stalk cell, and a head of 2–4 cells, where the length of the stalk was more than half the height of the head (Fig. 3d, e). PGTs and CGTs both possess a storage cavity, in which secreted metabolites often accumulate. The cavity can be subcuticular, where molecules secreted at the top of gland cells accumulate under the cuticle, which is gradually pushed away from the cell wall, as seen in *Artemisia annua* GTs [24]. Non-glandular trichomes were (1) dendritic, with 4–6 arms branching off from the basal region (Fig. 3g, h), (2) present at a high density on leaves, sepals, and stems, and (3) variable in length.

**Fig. 4:**
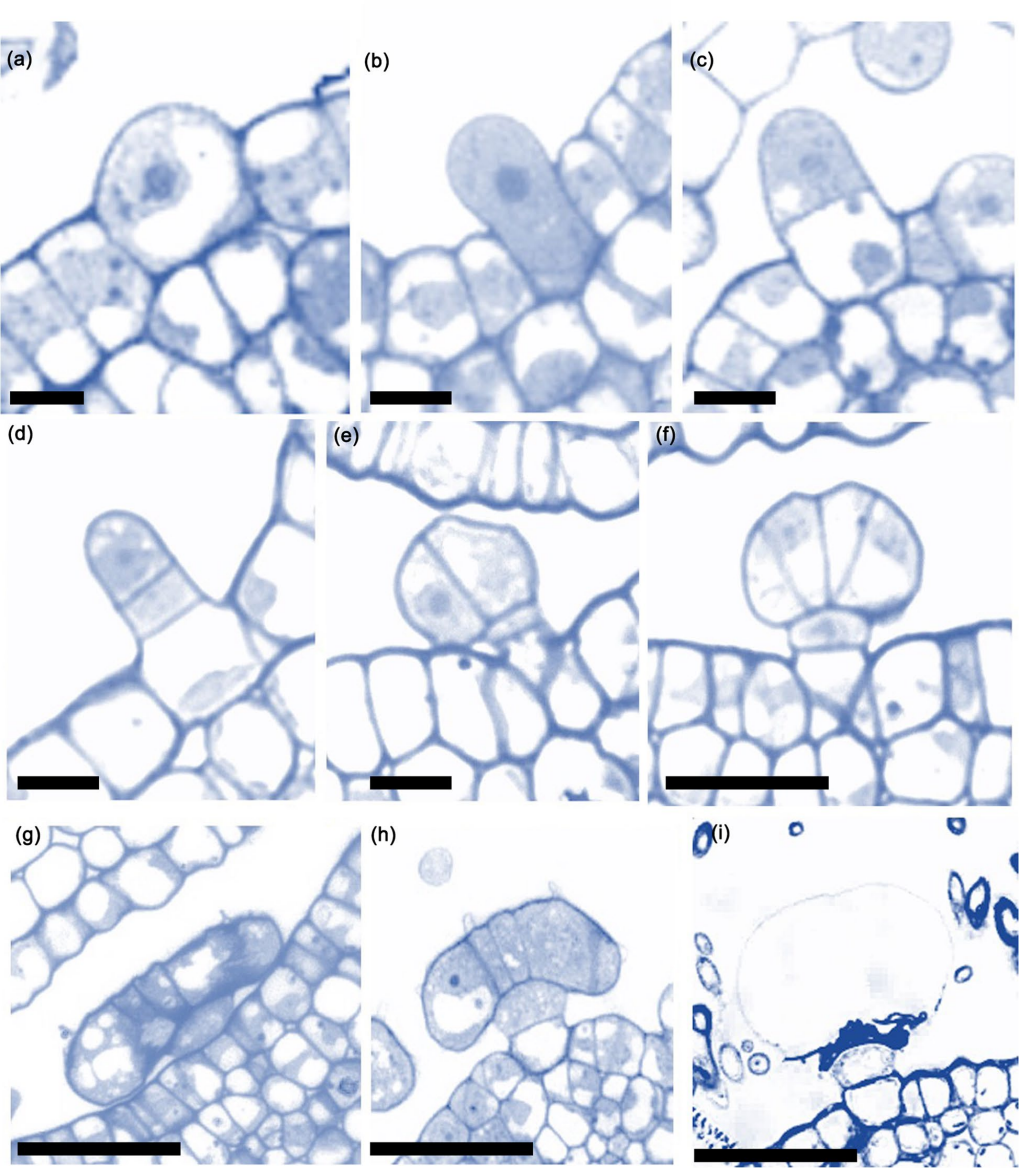
Characteristics of glandular trichomes (GTs) at different developmental stages (LM, light microscope). **a** Epidermal cells of leaves protrude, resulting in the formation of GT primitive cells at the early stage of development. **b–g** Cross-section of leaves with immature GT. **h** Cross-section of leaves with mature GT. **i** Cross-section of leaves with mature GT wrapped in a cuticle. Scale bars: 50 μm (**a**–**e**), 100 μm (**f**–**i**)

Next, we examined the developmental process of PGTs. Trichome initiation occurs when an epidermal cell acquires trichome identity upon receiving the signals from the surrounding cells. A PGT originated from a round protodermic cell, grew to the same size as the adjacent PGTs, and underwent periclinal division, forming a basal and an apical cell (Fig. 4a, b) and initiating a peduncle cell and an initial head cell (Fig. 4c). After the elongation period, the head cell underwent anticlinal division (Fig. 4d) and gave rise to two larger cells (Fig. 4e). Subsequently, the cells underwent successive anticlinal divisions (Fig. 4f, g) until a complete head was formed, which marked the end of the development (Fig. 4h, i). After the development of the PGT was complete, the secretory stage of the PGT was initiated. Microscopy analysis of the longitudinal section of a PGT showed that apical cells contained a dense cytoplasm (Fig. 4f–h), indicating that these apical cells will exhibit secretory activity. Within a PGT, essential oils are produced by the secretory apical cells, eight of which are arranged radially in a plane forming a subcuticular cavity above them.

### Comparison of the number and diameter size of PGTs among the four lavender varieties

**Fig. 5:**
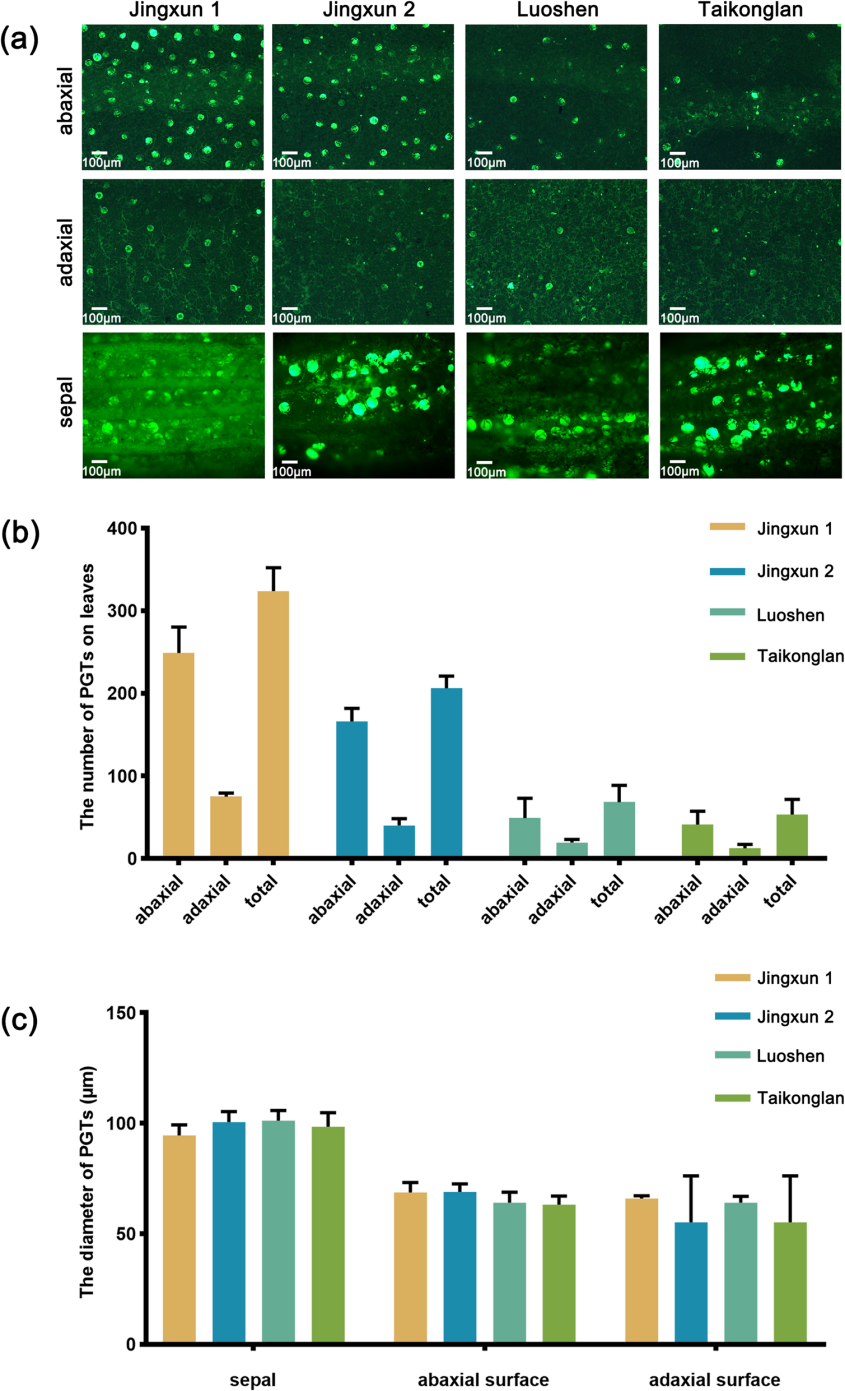
Analysis of the PGTs of four lavender cultivars. **a** Fluorescence microscopy images of the abaxial and adaxial leaf surfaces and sepals. **b** Number of PGTs on leaves. **c** Diameter of PGTs found on sepals and abaxial and adaxial leaf surfaces

We used a fluorescence microscope to observe GTs on the leaves and flowers of four lavender varieties. The number of PGTs differed significantly among the different organs of a variety and among the different varieties (Fig. 5). The number of PGTs was higher on the leaves of ‘Jingxun 1’ and ‘Jingxun 2’ than on the leaves of ‘Luoshen’ and ‘Taikonglan’, especially on the abaxial surface (Fig. 5a). The total number of PGTs on leaves was highest in ‘Jingxun 1’ and lowest in ‘Taikonglan’. Regardless of the variety, the number of PGTs on the abaxial surface of leaves was often greater than that on the adaxial surface of leaves (Fig. 5b). These results indicate that trichome density is variety- and plant organ-specific. The PGTs on sepals showed a larger diameter than those on leaves; however, the diameter of PGTs on sepal or leaf showed no significant difference among the four lavender varieties (Fig. 5c). The diameter of PGTs found on sepals varied from 91.86 to 109.32 μm; the smallest diameter was approximately 54.41 μm (on the adaxial surface), and the largest diameter was approximately 109.32 μm (on the sepal) (Supplementary Table S2). These results suggest that lavender essential oil is mainly derived from flowers.

### Identification and characterization of R2R3-MYB protein-coding genes involved in plant tissue development

**Fig. 6:**
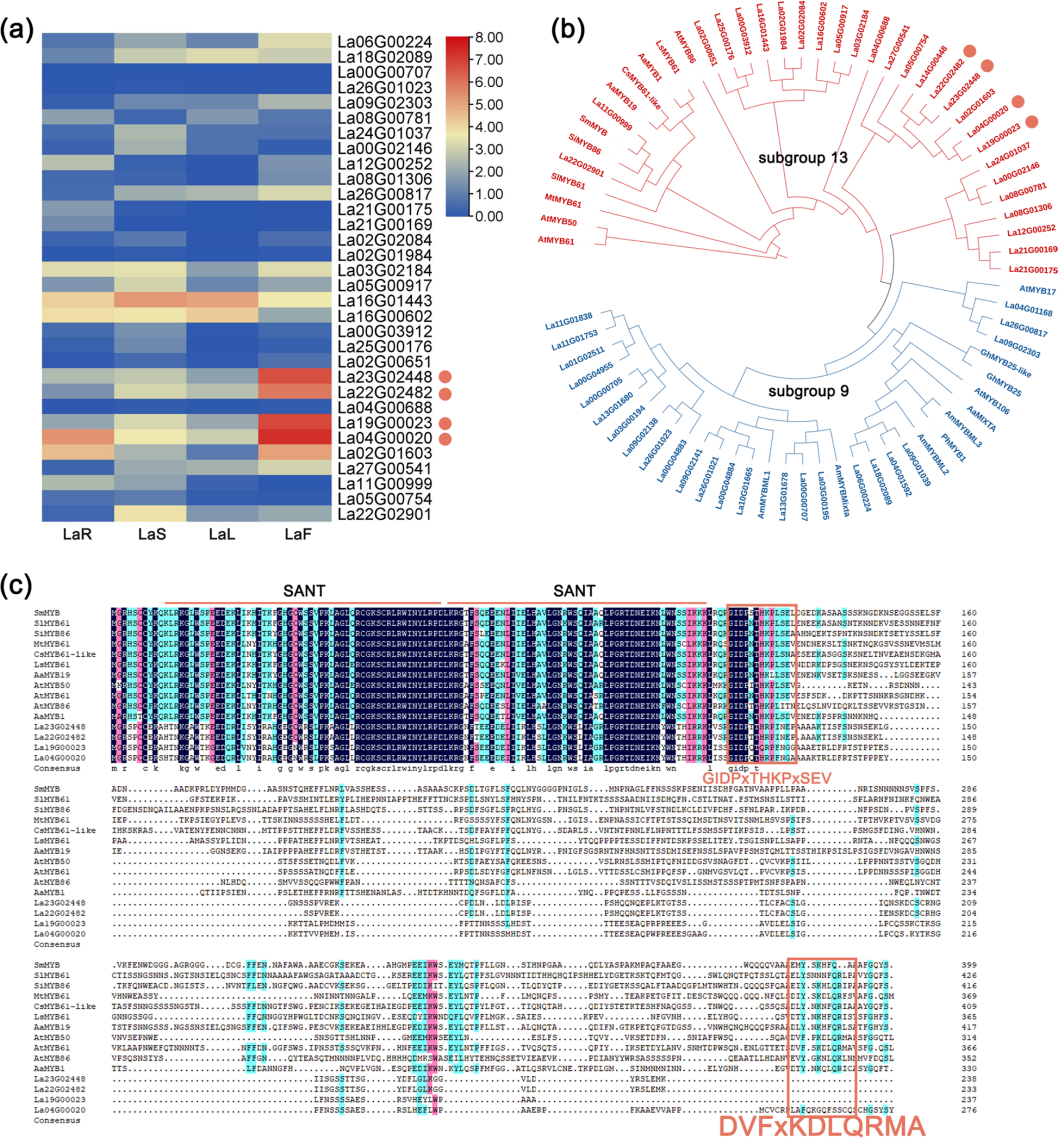
Characterization of R2R3-MYB protein-coding genes related to GT development. **a** Heatmap of R2R3-MYB genes in lavender. **b** Phylogenetic tree of genes included in R2R3-MYB subgroup 9 and subgroup 13 in lavender. **c** Multiple sequence alignment of four genes highly expressed in the flowers of lavender

We screened the ‘Jingxun 2’ genome database [5], and identified 51 R2R3-MYB proteins by performing a BLASTP search against the R2R3-MYB proteins of *Arabidopsis thaliana*, *Artemisia annua*, *Antirrhinum majus*, *Camellia sinensis*, *Gossypium hirsutum*, *Lactuca sativa*, *Medicago truncatula*, *Petunia hybrida*, *Salvia miltiorrhiza*, and *Solanum lycopersicum*. Based on the results of BLASTP analysis as well as transcriptome database search [25], we identified four genes, *La23G02448*, *La22G02482*, *La19G00023*, and *La04G00020*, which showed high expression levels in flowers (Fig. 6a). Phylogenetic analysis (Fig. 6b) showed that these four genes clustered with *AaMYB1* and *AtMYB61*, which have been reported to function in terpenoid metabolism and trichome development in *A. annua* and *A. thaliana* [26]. Further analysis indicated that these four genes clustered with the *MYB* genes of other plant species. Therefore, these genes were selected as candidates for further investigation.

Alignment of the deduced amino acid sequences of all genes that clustered with the four *L. angustifolia* genes revealed high sequence conservation at the N-terminus within the two MYB repeat domains, which are known to be important for DNA binding (Fig. 6c). The presence of the signature domain ‘GIDP_X_THKP_X_SEV’ or ‘DVF_X_KDLQRMA’ demonstrated that these proteins belong to the subgroup 13 of R2R3-MYB family [27]. According to previous studies, *AtMYB61* plays a pleiotropic role in regulating lignin deposition [28], mucilage production [29], and stomatal aperture [30], while *AtMYB1* and *AtMYB61* induce trichome initiation and branching in *A. thaliana* [26]. Therefore, we speculated that these four candidate genes may be related to cell fate regulation, similar to the genes belonging to subgroup 9.

## Discussion

VOCs constitute a large and diverse category of plant secondary metabolites, and function mainly to protect plants from herbivores and pathogenic microorganisms [31–33]. Terpenoids perform different biological activities [34]. Multiple studies have indicated that monoterpenoids are generally more abundant than sesquiterpenoids in *Chrysanthemum indicum* germplasm, regardless of the geographical origin [35–39]. Consistently, monoterpenoids were the most abundant compounds in lavender. Additionally, flowers were the main site of VOC accumulation in the four lavender varieties examined in this study. Plants synthesize an arsenal of terpenoid compounds that enhance defense against pathogens or herbivores, and flowering plants, in particular, contain an unusually high number of terpenoids [40]. These findings are consistent with the results of our study. The previous study showed that plant species with inflorescences bearing sequentially-opening flowers exhibit a long flowering life and lasting attraction for bees. Numerous and diverse volatiles emitted by flowers act as long-distance signals and as important cues for attracting pollinators [41, 42]. Together, these evidence indicate that flowers are the major organs that release volatile chemicals.

It has been reported that the initial cells of GTs originate from the epidermis. After their production, the meristematic cells protrude and divide into basal and apical cells, and the latter then divide into stalk and head cells [43, 44]. Different stem cell shapes lead to the formation of different types of GTs. Research on tomato, cucumber, sweet wormwood, tobacco, and cotton has significantly advanced our understanding of GT morphology and development [16]. In *A. annua*, GTs are composed of two basal cells, two stem cells, four near-head cells, and two head cells, all of which are arranged in a double row [45]. The morphogenetic process of GTs and trichomes has been elucidated in *Artemisia argyi*, which possesses two types of GTs: GT-I, which is composed of 10 biserial cells, and GT-II, which is composed of a row of 5 cells [46]. Although the types of GTs differ between these two *Artemisia* species, the pattern of GT development is the same. The pattern of PGT initiation has been defined in the leaves of peppermint (*Mentha* × *piperita*), which also belongs to the Lamiaceae family. Our results showed that PGTs in lavender are composed of an unicellular stalk, an unicellular base, and multiple 8-celled heads, each one of which assumes a globular dome shape at maturity, which is in agreement with the findings reported in peppermint [47]. In numerous Lamiaceae species, PGTs and small CGTs are formed on the leaves and bracts, while large CGTs are formed on the calyx [48]. CGTs likely exhibit a similar differentiation process as PGTs, but there are some differences. In *Millingtonia hortensis*, PGTs consist of a 12–24-cell disk-shaped head and a single-celled neck, while the CGTs are composed of a 4–8-cell head, single-celled neck, and a wide multicellular stalk. CGTs also differ from the PGTs by the presence of a wide stalk containing 2–3 layers of vacuolated cells [49]. In *Perilla frutescens*, the secretions of mature PGTs are yellow, and the diameter of PGTs differs from that of CGTs [17]. In *Mentha*, PGTs are composed of a basal cell, a short stalk cell, and 8–16 secretory cells, while the CGTs are of three types: type I CGTs possess a basal cell, a short stalk cell, and a large unicellular secretory head; type II CGTs contain a basal cell, a long stalk cell, and a smaller unicellular secretory head; and type III CGTs are composed of a large basal cell, an unicellular stalk cell, a thin neck cell, and a very small secretory head [50]. Therefore, in lavender, we speculate that CGTs do not represent a developmental stage of PGTs. Trichomes of several types can also exist on the same tissue. In this study, we found three types of trichomes on leaves, flowers, bracts, and calyx in lavender (Fig. 2). These results were the same as those reported in thyme, whose GTs were located mainly on leaves and flowers [20].

**Fig. 7:**
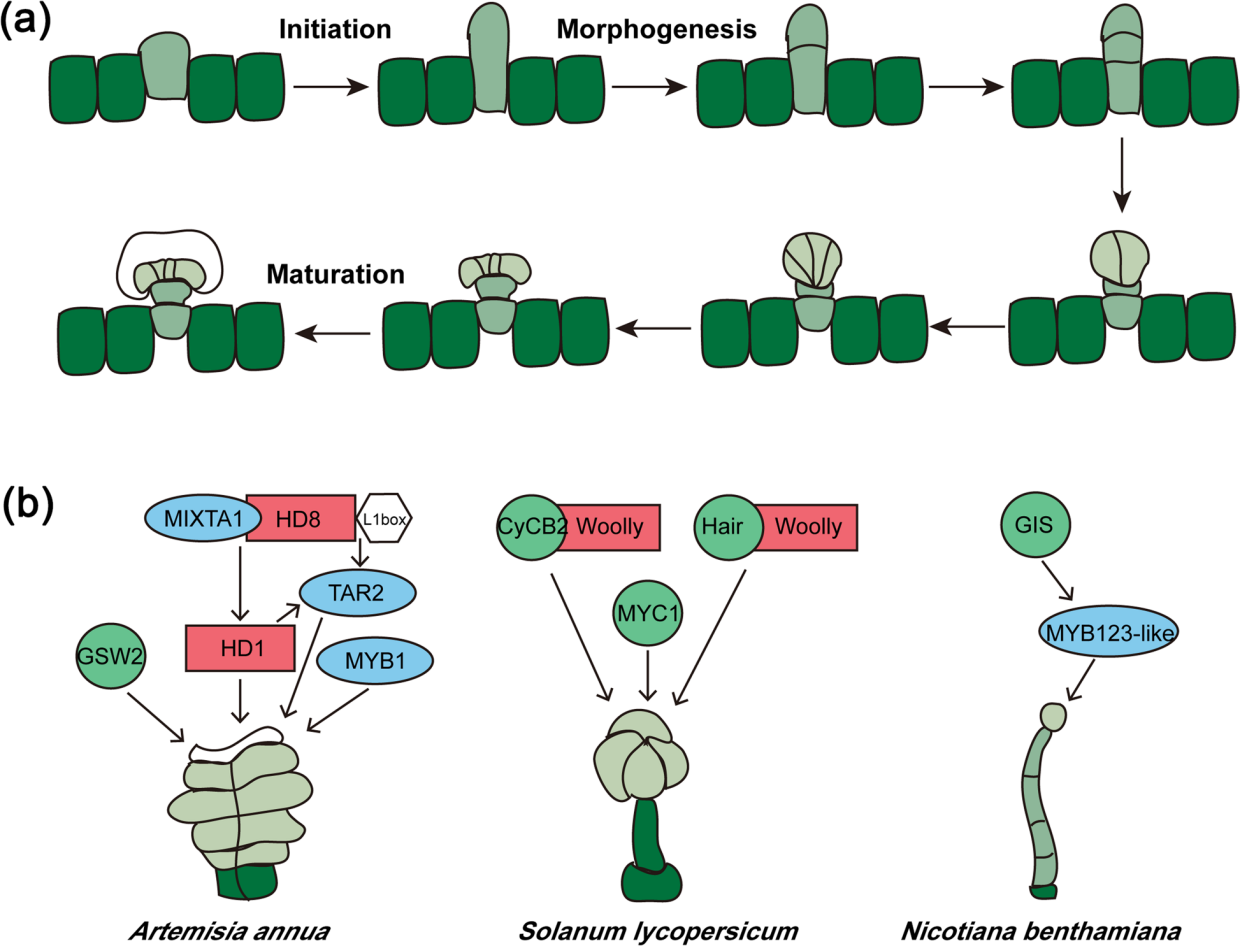
Model of GT development. **a** PGT development in lavender. **b** Genes involved in trichome development. Blue oval represents R2R3-MYB transcription factors; red box represents HD-ZIP IV transcription factors; green circle represents other genes

GT initiation is a complex process (Fig. 7a). A differentiating protodermal cell integrates both environmental and endogenous signals. Molecular data pointing toward genes that play a specific role in glandular trichome development already exist, especially concerning some transcription factors, cell cycle regulators, as well as receptors involved in phytohormone-induced signaling cascades (Fig. 7b). Some genes involved in the development of GTs have been reported in Lamiaceae species; for example, *TTG*1, *GTL*1, and *GL1* in basil, *MIXTA*-like and *HD1*-like in perilla, *StHD1* and *StHD8* in *Schizonepeta tenuifolia*, and *McMIXTA* and *McHD-Zip3* in mint [51–53]. However, most of these genes have not been investigated further (Table 2). Some genes related to GT development have been identified in other species and have been shown to form a regulatory network. For example, in sweet wormwood, *TAR2* regulates GT morphogenesis; in *A. annua*, *AaMYB1*, *AaMIXTA1*, and *AaHD1* directly regulate the formation of GTs, and AaMIXTA1 forms a complex with AaHD8 to promote *AaHD1* expression and positively regulate the initiation of GTs [54–56]; in tomato, *SlMYC1* regulates type VI formation, and *SlCycB2* and *Hair* interact with *Woolly* to regulate GTs [57–59]; and in tobacco, *NbGIS* regulates trichome initiation and acts upstream of *NbMYB123-like* [60], whereas NbCyCB2 inhibits trichome initiation by binding to the LZ domain of *NbWo* [61]. To sum up, GT development related genes in Lamiaceae species share high sequence homology with functionally similar genes in other plant species. In this study, we did not focus on the negative regulators of GTs.

**Table 2:** Genes involved in the development of glandular trichomes (GTs)

Family	Species	Gene name	Protein function	Protein family	Interacting protein	Reference
Lamiaceae	*Ocimum basilicum*	*TTG1*	Trichome development and information	WD40 repeat protein	MYC1	[51]
		*GTL1*	Trichome development and information	DNA-binding protein	-	
		*GL1*	Trichome development	MYB	-	
		*GIS2*	Trichome development	C2H2	-	
		*CPC*	Trichome development	MYB	-	
		*ETC3*	Trichome development	MYB	TRY, CPC3	
		*GL3*	Trichome development	bHLH	-	
		*TT8*	Trichome development	bHLH	-	
	*Perilla frutescens*	*MIXTA-like*	Trichome development	MYB	-	[17]
		*HD1-like*	Trichome development	HD-ZIP	-	
	*Schizonepeta tenuifolia*	*StHD1*	GT density and length	HD-ZIP	-	[52]
		*StHD8*	GT density and length	HD-ZIP	-	
	*Nepeta tenuifolia*	*HDG11*	Trichome	HD-ZIP	-	
	*Mentha canadensis*	*McMIXTA*	PGT development	MYB	McHD-Zip3	[53]
		*McHD-Zip3*	GT development	HD-ZIP	McMIXTA	
Asteraceae	*Artemisia annua*	*AaTAR2*	Trichome development	MYB	AaHD1, AaHD8	
		*AaMYB1*	GT density	MYB	-	[54]
		*AaMIXTA1*	GT density	MYB	Cuticle biosynthesis genes	[55]
		*AaHD1*	GT density	HD-ZIP	TAR2	[56]
		*AaHD8*	GT density	HD-ZIP	AaMIXTA1	
		*AaGSW2*	GT density	WRKY	AaHD1, AaHD8	
Solanaceae	*Solanum* *lycopersicum*	*SlMYC1*	Type VI formation	bHLH	TPSs	[57–59]
		*SlCycB2*	GT density	Cyclin	-	
		*Hair*	Type I formation	ZFPs	Woolly	
		*Woolly*	Type I density	HD-ZIP	SlCyB2	
	*Nicotiana tabacum*	*NbGIS*	GT density	ZFPs	-	[60, 61]
		*NbMYB123-like*	GT density	MYB	-	

## Conclusions

In this study, we explained the GT formation mechanism in lavender. The PGTs in lavender varieties were composed of a base, head, and stalk, and the head cells function to secrete VOCs. Further developmental analysis of PGTs is needed to understand whether the stalk cell develops a fully cutinized lateral wall or the large lipid inclusions accumulate in the secretory cells and stalk cell. In the future, we plan to perform a series of histochemical tests to examine the chemical composition in PGTs and determine the presence of acid polysaccharides, terpenes, phenolic substances, and other compounds.

We also compared the number and diameter size of PGTs among the four lavender varieties included in this study, and found that the numbers of PGTs on the abaxial and adaxial surfaces of leaves were higher in ‘Jingxun 1’ and ‘Jingxun 2’ than in ‘Luoshen’ and ‘Taikonglan’. In addition, we identified 66 VOCs in these four lavender cultivars, among which linalyl acetate and linalool were the most prominent. Our results indicated that flowers are the main site of accumulation of VOCs in lavender. Additionally, we identified four *R2R3-MYB* family genes, including *La23G02448*, *La22G02482*, *La19G00023*, and *La04G00020*, as candidates involved in GT development in lavender. This study provides new insights into the morphology of GTs. Further research is required to reveal the regulatory networks involved in the formation of GTs in lavender.

## Methods

### Plant material

Lavender cultivars ‘Jingxun 1’, ‘Jingxun 2’, ‘Luoshen’, and ‘Taikonglan’ were used in this study. ‘Jingxun 1’, ‘Jingxun 2’, and ‘Luoshen’ were bred by our research team, and ‘Taikonglan’ is a commercial variety maintained by our research team. All four varieties were cultivated at the Institute of Botany, CAS, Beijing, China.

### HS-SPME procedure

To perform HS-SPME, approximately 0.10 g of flower samples and 0.15 g of leaf samples were weighed and immediately placed into a 20-ml head-space vial (Agilent, Palo Alto, CA, USA) containing 20 μl of 3-Octanol (7.5 mg/ml; internal standard) (Aladdin, Shanghai, China; Cas#589–98-O). The vials were sealed using crimp-top caps with PTFE-silicone headspace septa (Agilent, Palo Alto, CA, USA). Subsequently, each vial was immediately incubated at 65℃ for 30 min. Then, 100-μm divinylbenzene/carboxen/polydimethylsiloxane coating fiber (Supelco, Inc., Bellefonte, PA, USA) was exposed to the headspace for 30 min at 65℃ to absorb the volatiles, split ratio was 70:1. All the volatile components on the coating fiber were then analyzed by gas chromatography.

### GC–MS analysis

A Model 7890B GC instrument (Agilent, Palo Alto, CA, USA) and a 7000D mass spectrometer (Agilent, Palo Alto, CA, USA) were used to perform GC–MS analysis. Briefly, after microextraction, the desorption of VOCs from the coating fiber was performed in the injection port of the GC apparatus at 250℃ for 4 min in the splitless mode. A DB-5MS (5% phenyl-polymethylsiloxane, 30 m × 0.25 mm × 1.0 μm) capillary column was used to identify and quantify the VOCs. Helium (99.999% purity) was used as the carrier gas at a flow rate of 1.0 ml/min. The front injector temperature was kept at 250℃, and the detector was kept at 280℃. The column temperature was set at 60℃ for 1 min and then raised to 240℃ at a rate of 5℃/min. The temperature of the transfer line, ion source, and quadrupole mass detector was set at 280℃. Mass spectra were recorded in electron impact (EI) ionization mode at 70 eV and were scanned at 1-s intervals at a mass-to-charge (m/z) ratio ranging from 30–500 amu [62].

### VOC identification and quantification

Volatile compounds were identified by comparing the mass spectra of the published standard substance retaining index as well as information from the National Institute of Standards and Technology (NIST) [63]. The original data acquired by GC–MS were first deconvoluted using Masshunter Agilent Analysis (B.08.00; Agilent, Palo Alto, CA, USA) to obtain the retention time, peak area, and m/z ratio of characteristic peaks. Internal standards were used to normalize the data. Each chromatographic peak area represented the relative content of the corresponding substance. Finally, the integral data of all chromatographic peak areas were exported for statistical analysis.

### Stereomicroscopy and fluorescence microscopy

Fresh flowers and leaves were collected to observe trichomes using a stereomicroscope (Leica DVM6) and fluorescence microscope (Leica DM6 B). Because the plant materials were three-dimensional, several photographs were taken in the layer scan mode and then assembled to synthesize one image.

### Scanning electron microscopy (SEM)

Fresh tissues were collected and cut into approximately 5-mm^2^ pieces, which were then immediately transferred into the FAA solution (50% ethanol: glacial acetic acid: methyl aldehyde = 90:5:5, v/v/v) and incubated at 4℃ for 24 h. The samples were dehydrated using an ethanol series (70%, 80%, 90%, 95%, and 100%) for 15 min, and then subjected to carbon dioxide critical point drying. The dried samples were glued on to an SEM stub and gold-coated before imaging in the main chamber of the microscope under high-vacuum and room-temperature conditions.

### Light microscopy

Leaves at different developmental stages were fixed at 4℃ for 6 h in 0.1 M phosphate buffer containing 1.5% (v/v) glutaraldehyde, 2% (v/v) paraformaldehyde, and 2% sucrose. The fixed leaf samples were post-fixed in osmium tetroxide, washed with phosphate buffer, dehydrated using an acetone series, and finally embedded in Spurr’s resin. Thin sections were prepared using a LEICA UC6I microtome [64].

### PGT counting

Fully-expanded mature leaves were collected from the plants grown under the same conditions as per replicate. Three different leaves at the same position were collected from each independent plant. The PGT count was determined from micrographs using the ImageJ software (http://rsb.info.nih.gov/ij), as reported previously [65].

### Statistical analysis

All samples were prepared and analyzed in triplicate, and data were expressed as the mean ± SD. Statistical analyses were performed by analysis of variance (ANOVA), and Duncan’s test was used to determine the significance of differences between groups.

## Supplementary Information

**Additional file 1:** **Fig. S1.** Pictures of sepals (dried) from four lavenders by stereoscope. **Fig. S2.** Pictures of stem, abaxial and adaxial of leaves surface by stereoscope. **Fig. S3.** Pictures of peltate trichome by SEM. **Table S1.** Number of peltate glandular trichomes of four lavender varieties. **Table S2.** Diameter size of PGTs of four lavender varieties.

## Data Availability

The raw whole-genome and transcriptome sequencing data used in this study are available from the National Center for Biotechnology Information (NCBI) database (Project No. PRJNA642976). The data generated and material used in the current study are available from the corresponding author upon reasonable request.
