# Supplementary material for: Formation mechanism of glandular trichomes involved in the synthesis and storage of terpenoids in lavender
Source: BMC Plant Biol. 2023 Jun 8;23:307. doi: 10.1186/s12870-023-04275-y (PMC10249152; doi:10.1186/s12870-023-04275-y)
Supplement: Supplementary file 1 — Additional file 1: Fig. S1. Pictures of sepals (dried) from four lavenders by stereoscope. Fig. S2. Pictures of stem, abaxial and adaxial of leaves surface by stereoscope. Fig. S3. Pictures of peltate trichome by SEM. Table S1. Number of peltate glandular trichomes of four lavender varieties. Table S2. Diameter size of PGTs of four lavender varieties. [file 12870_2023_4275_MOESM1_ESM.docx]

**Supplementary Information**

**Additional file 1: Supplementary Fig. S1.** Pictures of sepals (dried) from four lavenders by stereoscope.

**Additional file 2: Supplementary Fig. S2.** Pictures of stem, abaxial and adaxial of leaves surface by stereoscope.

**Additional file 3: Supplementary Fig. S3.** Pictures of peltate trichome by SEM.

**Additional file 4: Supplementary Table S1.** Number of peltate glandular trichomes of four lavender varieties.

**Additional file 5: Supplementary Table S2.** Diameter size of peltate glandular trichomes of four lavender varieties.

**Supplemental Figures and Supplementary Tables**


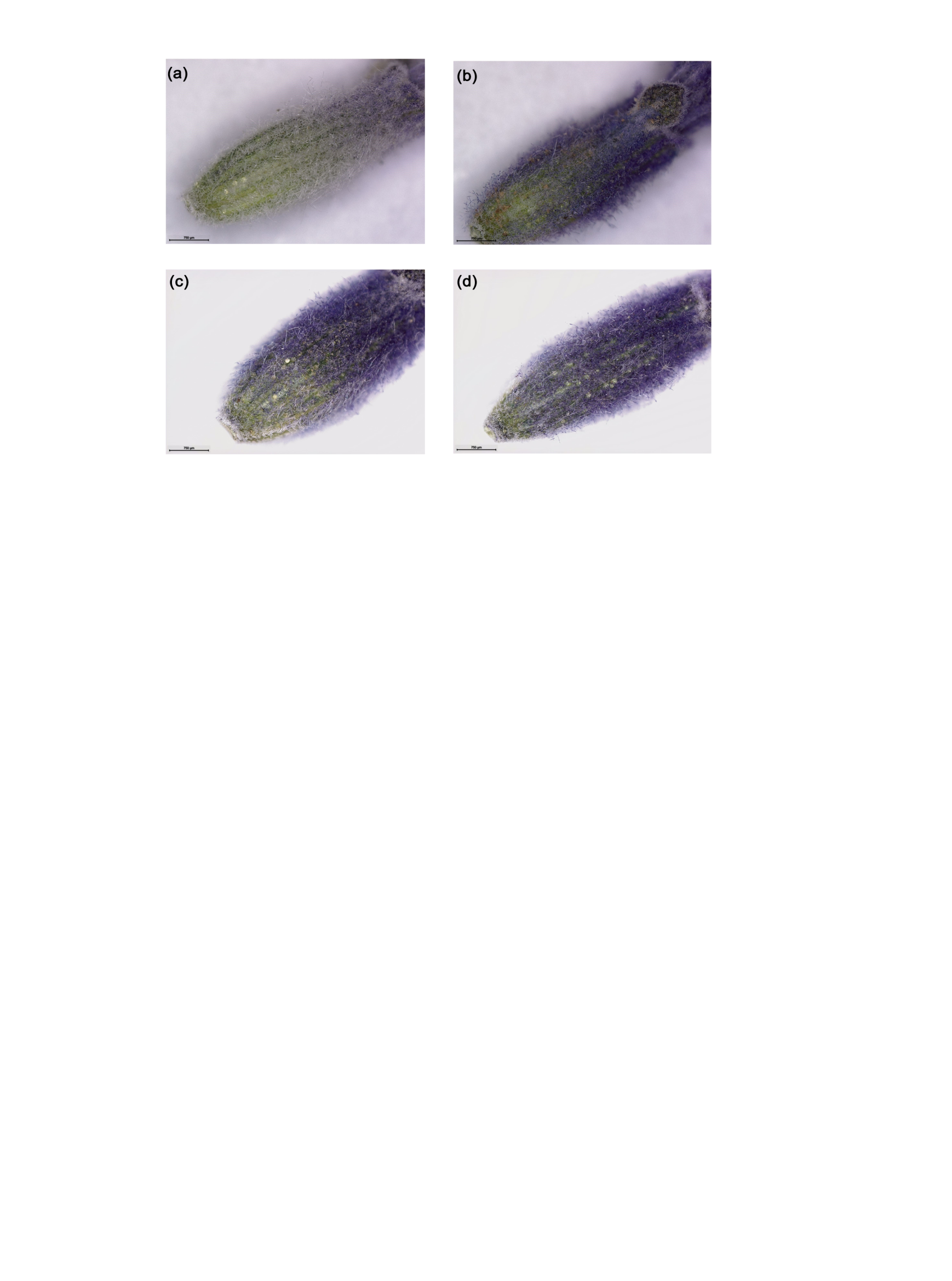


**Fig. S1** **Pictures of sepals (dried) from four lavenders by stereoscope. (a)** ‘Jingxun 1’ sepal. **(b)** ‘Jingxun 2’ sepal. **(c)** ‘Luoshen’ sepal. **(d)** ‘Taikonglan’ sepal.


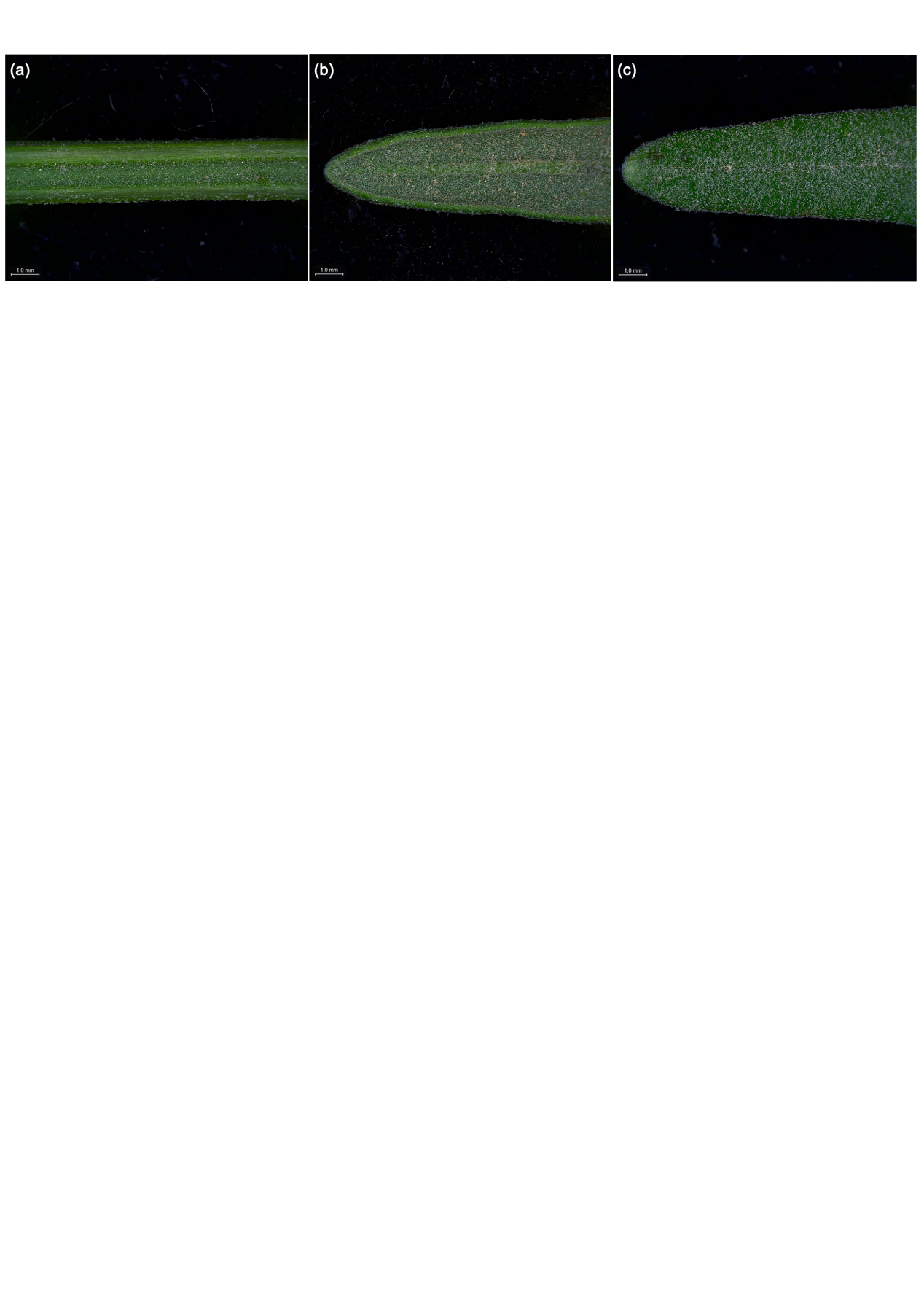


**Fig. S2** **Pictures of stem, abaxial, and adaxial of leaf surfaces by stereoscope**. **(a)** Stem. **(b)** Abaxial surface. **(c)** Adaxial surface.


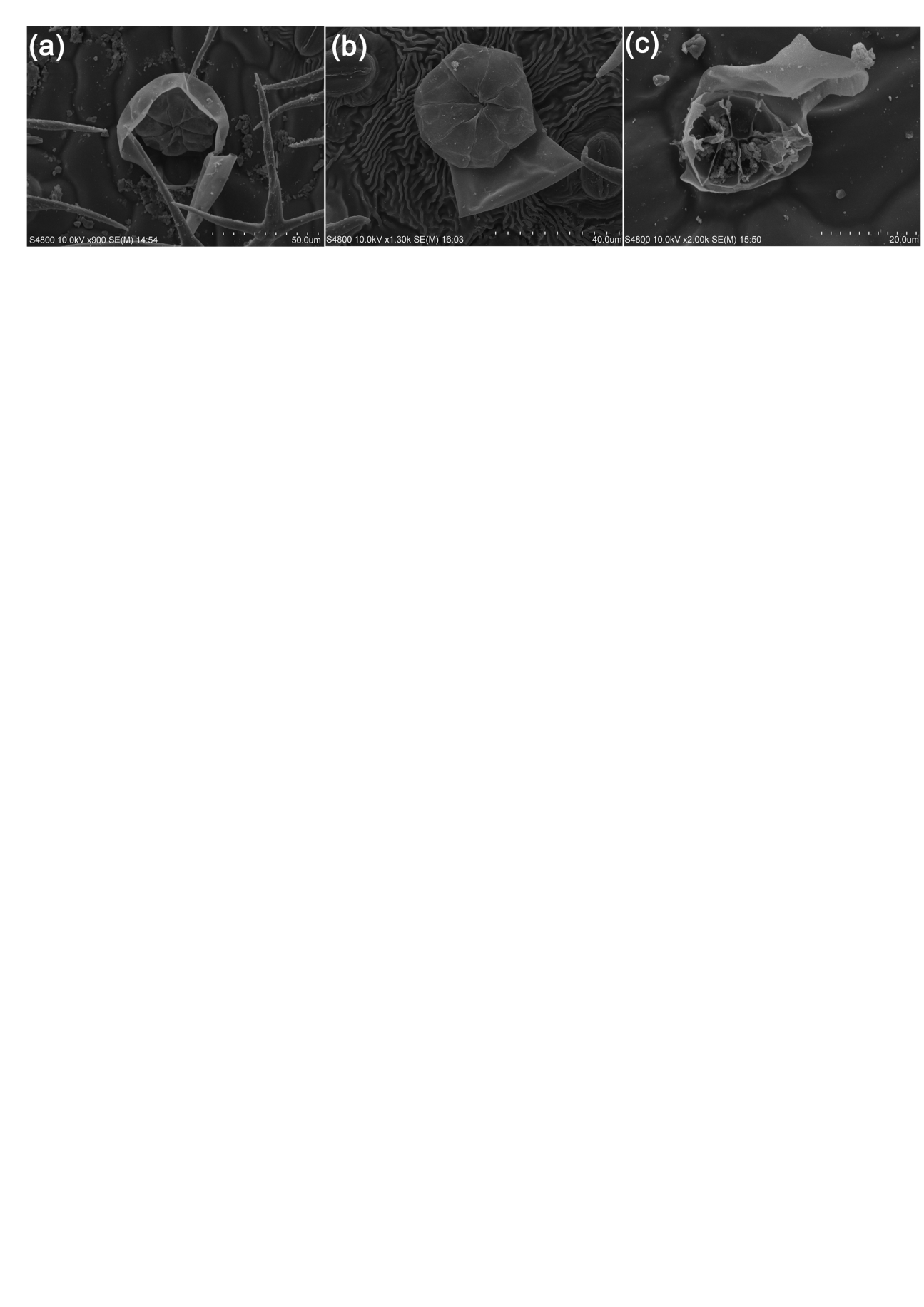


**Fig. S3** **Pictures of peltate trichome by SEM**. **(a)** Peltate trichome of broken cuticle. **(b)** Peltate trichome with complete broken cuticle. **(c)** Inclusion release in peltate trichome.

**Supplementary Table S1.** **Number of peltate glandular trichomes of four lavender varieties.**

| **Lavender varieties** | **Abaxial surface** | **Adaxial surface** | **Total** |
| --- | --- | --- | --- |
| **Jingxun 1** | 233 | 80 | 313 |
| **Jingxun 1** | 228 | 74 | 302 |
| **Jingxun 1** | 285 | 71 | 356 |
| **Jingxun 2** | 155 | 35 | 190 |
| **Jingxun 2** | 184 | 35 | 219 |
| **Jingxun 2** | 159 | 50 | 209 |
| **Luoshen** | 77 | 15 | 92 |
| **Luoshen** | 33 | 22 | 55 |
| **Luoshen** | 37 | 21 | 58 |
| **Taikonglan** | 58 | 16 | 74 |
| **Taikonglan** | 25 | 14 | 39 |
| **Taikonglan** | 40 | 7 | 47 |

| **Lavender varieties** | **Sepal (****μm)** | **Abaxial surface (μm)** | **Adaxial surface (μm)** |
| --- | --- | --- | --- |
| **Jingxun 1** | 91.89 | 75.24 | 67.04 |
| **Jingxun 1** | 97.98 | 64.89 | 67.38 |
| **Jingxun 1** | 105.38 | 70.37 | 65.9 |
| **Jingxun 1** | 89.69 | 63.74 | 64.12 |
| **Jingxun 1** | 94.18 | 66.43 | 66.22 |
| **Jingxun 1** | 95.65 | 65.58 | 65.12 |
| **Jingxun 1** | 92.4 | 67.91 | 66.17 |
| **Jingxun 1** | 92.03 | 67.91 | 64.42 |
| **Jingxun 1** | 91.86 | 76.44 | 67.15 |
| **Jingxun 2** | 104.2 | 74.02 | 54.41 |
| **Jingxun 2** | 100.09 | 70.61 | 56.36 |
| **Jingxun 2** | 97.48 | 65.21 | 62.29 |
| **Jingxun 2** | 98.18 | 70.1 | 57.54 |
| **Jingxun 2** | 107.34 | 68.05 | 61.33 |
| **Jingxun 2** | 106.38 | 63.58 | 65.05 |
| **Jingxun 2** | 92.32 | 73.85 | 64.57 |
| **Jingxun 2** | 99.87 | 66.61 | 68.91 |
| **Jingxun 2** | 99.05 | 68.62 | 62.04 |
| **Luoshen** | 102.11 | 67.9 | 62.52 |
| **Luoshen** | 104.2 | 65 | 67.37 |
| **Luoshen** | 109.32 | 65.79 | 65.77 |
| **Luoshen** | 103.14 | 57.47 | 67.48 |
| **Luoshen** | 95.96 | 67.19 | 63.34 |
| **Luoshen** | 103.14 | 58.66 | 59.13 |
| **Luoshen** | 99.52 | 71.01 | 66.51 |
| **Luoshen** | 93.86 | 58.75 | 62.17 |
| **Luoshen** | 99.52 | 65.04 | 62.16 |
| **Taikonglan** | 92.82 | 67.47 | 57.12 |
| **Taikonglan** | 105.29 | 61.32 | 57.12 |
| **Taikonglan** | 105.77 | 56.63 | 56.91 |
| **Taikonglan** | 104.77 | 66.46 | 65.36 |
| **Taikonglan** | 100.1 | 68.06 | 63.76 |
| **Taikonglan** | 91.88 | 63.76 | 64.73 |
| **Taikonglan** | 90.33 | 58.82 | 57.56 |
| **Taikonglan** | 101.62 | 64.04 | 65.42 |
| **Taikonglan** | 92.88 | 61.86 | 65.74 |

**Supplementary Table S2.** **Diameter size of PGTs of four lavender varieties.**
